# Factors associated with resolution of type-2 diabetes mellitus after sleeve gastrectomy in obese adults

**DOI:** 10.1038/s41598-021-85450-9

**Published:** 2021-03-16

**Authors:** Ahmed Abdallah Salman, Mohamed Abdalla Salman, Mohamed A. Marie, Ahmed Rabiee, Mona Youssry Helmy, Mohamed Sabry Tourky, Mohamed Gamal Qassem, Hossam El-Din Shaaban, Mohamed D. Sarhan

**Affiliations:** 1 Internal Medicine Department, Faculty of Medicine, Cairo University, Cairo, Egypt; 2 General Surgery Department, Faculty of Medicine, Cairo University, Cairo, Egypt; 3 Department of Surgery, Great Western Hospitals NHS Foundation Trust, Swindon, UK; 4 Department of General Surgery, Faculty of Medicine, Ain Shams University, Cairo, Egypt; 5 Gastroenterology Department, National Hepatology and Tropical Medicine Research Institute, Cairo, Egypt

**Keywords:** Diseases, Endocrinology

## Abstract

Many bariatric procedures are more effective for improving type-2 diabetes mellitus (T2DM) than conventional pharmacotherapy. The current research evaluated factors linked to complete and partial remission or improvement of T2DM after laparoscopic sleeve gastrectomy (LSG). The current prospective study included all diabetic patients who were submitted LSG between January 2015 and June 2018 and completed a 2-year follow-up period. Patients were assessed at baseline and 2 years after LSG. This work comprised of 226 diabetic cases. Two years after LSG, 86 patients (38.1%) achieved complete remission of DM, and 24 (10.6%) reached partial remission. Only 14 patients (6.2%) showed no change in their diabetic status. On univariate analysis, age ≤ 45 years, duration of diabetes ≤ 5 years, use of a single oral antidiabetic, HbA1c ≤ 6.5%, HOMA-IR ≤ 4.6, C-peptide > 2.72 ng/mL, and BMI ≤ 40 kg/m^2^ predicted complete remission. The independent predictors of complete remission were age ≤ 45 years, duration of diabetes ≤ 5 years, use of a single oral antidiabetic, HOMA-IR ≤ 4.6, and C-peptide > 2.72 ng/mL. A combined marker of young age, short duration of DM, and low HOMA-IR predicted complete remission with sensitivity 93% and specificity 82%. Independent predictors of complete remission of T2DM after LSG were younger age, shorter duration, single oral antidiabetic, lower HOMA-IR, and higher C-peptide.

## Introduction

In 1997, obesity was declared an epidemic by the World Health Organization^1^; however, its prevalence is increasing worldwide. It is estimated that over 2 billion adults, i.e., about 39% of human beings, were overweight or obese in 2016^2^. The burden of obesity is aggravated by its associated comorbidities as type 2-diabetes mellitus (T2DM) and cardiovascular diseases (CVD), among others^3^. The increase in obesity rates seems to be the main factor for the recent surge in the prevalence of T2DM^4^. About 44% of the burden of T2DM is attributable to overweight and obesity^5^, and 9.5% of all body mass index-related deaths were due to diabetes in obese patients^4^.

Despite efforts through lifestyle interventions to treat obesity and T2DM, only a minority of patients achieved long-term weight loss and glycemic targets^6^. There is a reasonable body of evidence to confirm that many bariatric procedures are more effective than conventional pharmacotherapy for improvement or even inducing complete remission of T2DM^7–10^. These procedures include Roux-en-Y gastric bypass^11^, sleeve gastrectomy^12^, and duodenal switch/biliopancreatic diversion^13^.

Recently, laparoscopic sleeve gastrectomy (LSG) turned into the most popular bariatric operation in many parts of the world^14^. The current analysis aimed to assess the possible factors linked to complete and partial remission or improvement of T2DM after laparoscopic sleeve gastrectomy (LSG).

## Patients and methods

This prospective work included patients who were submitted LSG between January 2015 and June 2018. The ethics committee approved the study at the Cairo University Hospitals. All methods were performed in accordance with the relevant guidelines and regulations. Besides, written informed consent was obtained from all subjects.

Inclusion criteria were subjects with T2DM, aged 18–65 years, with BMI ≥ 35. Exclusion criteria were subjects who had revisional LSG and those with type-1 diabetes mellitus.

Patients were reviewed at baseline and 2 years after LSG. Data registered were demographic and anthropometric (age, sex, weight), laboratory (fasting blood glucose, HbA1c, C-peptide, insulin level), and clinical (insulin and/or oral medications, diabetes duration, the existence of elevated blood pressure, hyperlipidemia) characteristics. Percent weight loss and BMI change were obtained using the previously reported methods^15^. Insulin resistance was calculated by the homeostasis model assessment of insulin resistance (HOMA-IR)^16^.

The current analysis adopted the standardized American Society of Bariatric and Metabolic Surgery definitions of evolution of T2D post-bariatric procedures^17^ (Table 1).

**Table 1:** Determination of the status of type 2 diabetes^17^.

Status	Description and criteria
Complete remission	Normal levels of glucose parameters (HbA1c < 6% and FBG < 100 mg/dL) without the use of hypoglycemic medications
Partial remission	Defined as HbA1c 6%–6.4% and FBG 100–125 mg/dL without the use of hypoglycemic medications
Improvement	Decrease in HbA1c and FBG not fulfilling criteria for remission, or reduction in antidiabetic drug use
Unchanged	Neither remission nor improvement

*HbA1c* Glycated hemoglobin; *FBG* fasting blood glucose.

The operation began with the division of gastrosplenic ligament along the greater curvature 4 cm from the pylorus up to the left diaphragmatic crus with ultrasonic shears^18^. The stomach was then mobilized and divided along the lesser curvature from antrum (4 cm from pylorus) till reaching the angle of His using buttressed (SeamGuard, Gore, Inc., Flagstaff, Arizona, USA) linear 60-mm stapler (Covidien Tristapler, Medtronic, Minneapolis, Minnesota, USA) over the calibration tube (Midsleeve 36 Fr) introduced into the gastric lumen^18^. The specimen was extracted through the umbilical port. The operation was ended with a methylene blue leak testing.

Statistical analysis was performed using IBM SPSS Statistics version 22 (IBM Corp., Armonk, NY, USA). The power of the test used for primary outcome measure was estimated using the G Power software (Institutfür Experimentelle Psychologie, Heinrich Heine Universität, Düsseldorf, Germany) version 3.1.9.2. Numerical variables were calculated as mean and standard deviation or median and range as appropriate. Qualitative variables were calculated as frequency and percentage. Chi-square test (Fisher’s exact test) was used to examine the relation between qualitative variables. For quantitative variables, comparison between two arms was made using independent sample t-test or Mann–Whitney test. Multivariate analysis was performed using logistic regression method for the significant factors found on univariate analysis. Odds ratio (OR) with its 95% confidence interval (CI) were used for risk estimation. A p-value < 0.05 was considered significant.

### Consent to participate

Consents were done for all enrolled cases.

## Results

At the start of the study, 254 patients were included, however, only 226 completed the follow-up. Only those who completed the follow-up were included in the analysis.

Table 2 shows the baseline data of the 226 patients of the studied group. Hypertension and dyslipidemia were common comorbidities. Near 40% of the studied group received insulin for glycemic control.

**Table 2:** Baseline clinical and laboratory data of the enrolled group.

	Value
Age (years)	41.6 ± 8.8
Sex (male/female)	128/98
Weight (kg)	128.7 ± 13.2
Body mass index (kg/m^2^)	43.1 ± 4.6
Duration of diabetes mellitus	6 (1–15)
Hypertension	114 (50.4%)
Dyslipidemia	98 (43.4%)
**Treatment of diabetes mellitus**	
Insulin therapy	86 (38.1%)
Number of oral hypoglycemic agents	2 (1–4)
Sulphonylurea	158 (69.9%)
Metformin	190 (84.1%)
Incretin mimics	92 (40.7%)
SGT2-inhibitor	40 (17.7%)
Pioglitazone	16 (7.1%)
**Laboratory findings**	
C-peptide (ng/dL)	3.8 ± 1.7
Fasting blood glucose (mg/dL)	172.6 ± 63.5
Glycated hemoglobin (%)	7.7 ± 1.2
Homeostatic Model Assessment of Insulin Resistance	4.6 (1.3–12.4)

Data are expressed as mean ± SD, median (range), or number (%).

Two years after LSG, 86 patients (38.1%) achieved complete remission of DM, and 24 (10.6%) reached partial remission. Besides, improvement of diabetes occurred in 102 patients (45.1%). Only 14 patients (6.2%) showed no change in their diabetic status. More than half of those with hypertension and dyslipidemia got resolution of the disease (Table 3).

**Table 3:** Outcome of sleeve gastrectomy after 2 years of follow up.

	Value
Weight (kg)	102.7 ± 10.5
Body mass index (kg/m^2^)	29.8 ± 5.1
Weight loss (kg)	26.0 ± 8.9
Percent weight loss	20.0 ± 5.8
Body mass index loss (kg/m^2^)	13.3 ± 2.0
Percent body mass index loss	31.1 ± 5.8
**Outcome of diabetes mellitus**	
Complete remission	86 (38.1%)
Partial remission	24 (10.6%)
Improvement	102 (45.1%)
No change	14 (6.2%)
Hypertension resolution	64 (56.1%)
Dyslipidemia resolution	52 (53.1%)
**Laboratory findings**	
Fasting blood glucose (mg/dL)	132.4 ± 52.7
Glycated hemoglobin (%)	6.5 ± 1.3
Homeostatic Model Assessment of Insulin Resistance	3.2 (1.0–10.4)

Data are expressed as mean ± SD, median (range), or number (%).

Table 4 shows a significant difference between patients who developed complete remission of T2DM and those who did not, in all pre-and postoperative characteristics except sex, hypertension, dyslipidemia, insulin therapy, and preoperative weight.

**Table 4:** Pre- and postoperative characteristics in cases who developed complete remission of T2DM 2 years after LSG and those who did not.

		CR n = 86	No CR n = 140	*p* value
Age (years)		36.4 ± 6.7	44.8 ± 8.4	< 0.001
Sex (male/female)	Male	50 (39.1%)	78 (60.9%)	0.721
	Female	36 (36.7%)	62 (63.3%)	
Duration of diabetes mellitus		4 (1–8)	7 (1–15)	< 0.001
Hypertension	Yes	46 (40.4%)	68 (59.6%)	0.473
	No	40 (35.7%)	72 (64.3%)	
Dyslipidemia	Yes	38 (38.8%)	60 (61.2%)	0.845
	No	48 (37.5%)	80 (62.5%)	
**Treatment of diabetes mellitus**				
Insulin therapy	Yes	26 (30.2%)	60 (69.8%)	0.058
	No	60 (42.9%)	80 (57.1%)	
No. of oral hypoglycemics	Single	36 (69.2%)	16 (30.8%)	< 0.001
	Multiple	50 (28.7%)	124 (71.3%)	
**Preoperative findings**				
Weight (kg)		130.8 ± 13.3	127.4 ± 13	0.058
BMI (kg/m^2^)		42.2 ± 4.5	43.6 ± 4.7	0.025
C-peptide (ng/mL)		5.3 ± 1.5	2.9 ± 1.1	< 0.001
Fasting blood glucose (mg/dL)		135.5 ± 40.6	195.4 ± 64.4	< 0.001
Glycated hemoglobin (%)		7.1 ± 1.0	8.1 ± 1.2	< 0.001
HOMA-IR		3.39 ± 1.23	6.18 ± 2.21	< 0.001
**Postoperative findings**				
Weight (kg)		97.2 ± 8.5	106.0 ± 10.2	< 0.001
BMI (kg/m^2^)		27.4 ± 4.3	31.3 ± 5.0	< 0.001
% Weight loss		25.5 ± 4.0	16.7 ± 3.8	< 0.001
% BMI loss		35.3 ± 4.4	28.5 ± 4.9	< 0.001
Fasting blood glucose (mg/dL)		84.4 ± 7.2	161.9 ± 46.5	< 0.001
Glycated hemoglobin (%)		5.3 ± 0.4	7.2 ± 1.2	< 0.001
HOMA-IR		2.2 ± .9	4.3 ± 1.5	< 0.001

*CR* complete remission, *BMI* body mass index, *HOMA-IR* homeostatic model assessment of insulin resistance.

On univariate analysis, predictive factors for complete remission were age ≤ 45 years, duration of diabetes ≤ 5 years, use of a single oral antidiabetic, HbA1c ≤ 6.5%, HOMA-IR ≤ 4.6, C-peptide > 2.72 ng/mL, and BMI ≤ 40 kg/m^2^ (Table 5). Using logistic regression, the independent predictors of complete remission were age ≤ 45 years, duration of diabetes ≤ 5 years, use of a single oral antidiabetic, HOMA-IR ≤ 4.6, and C-peptide > 2.72 ng/mL (Table 6). A combined marker of young age, short duration of DM, and low HOMA-IR provide a very good prediction of complete remission with sensitivity 93% and specificity 82%.

**Table 5:** Factors linked to complete remission of T2DM 2 years after LSG.

	CR n = 86	No CR n = 140	*p* value	OR (95% CI)
**Age (years)**				
≤ 45	76 (53.5%)	66 (46.5%)	< 0.001	8.52 (4.07–17.83)
> 45	10 (11.9%)	74 (88.1%)		1
**Duration of DM**				
≤ 5 years	70 (64.8%)	38 (35.2%)	< 0.001	11.74 (6.08–22.69)
> 5 years	16 (13.6%)	102 (86.4%)		1
**Insulin therapy**				
No	60 (42.9%)	80 (57.1%)	0.058	1.73 (0.98––3.06)
Yes	26 (30.2%)	60 (69.8%)		1
**No. of oral hypoglycemics**				
Single	36 (69.2%)	16 (30.8%)	< 0.001	5.58 (2.84–10.95)
Multiple	50 (28.7%)	124 (71.3%)		1
**Preoperative BMI**				
≤ 40 kg/m^2^	33 (47.8%)	36 (52.2%)	0.045	1.80 (1.01–3.20)
> 40 kg/m^2^	53 (33.8%)	104 (66.2%)		1
C-peptide (ng/mL)				
> 2.72	84 (56.8%)	64 (43.2%)	< 0.001	49.88 (11.80–210.76)
≤ 2.72	2 (2.6%)	76 (97.4%)		1
**Glycated hemoglobin (%)**				
≤ 6.5	22 (78.6%)	6 (21.4%)	< 0.001	7.68 (2.97–19.86)
> 6.5	64 (32.3%)	134 (67.7%)		1
**HOMA-IR**				
≤ 4.6	76 (67.9%)	36 (32.1%)	< 0.001	21.96 (10.26–45.97)
> 4.6	10 (8.8%)	104 (91.2%)		1

*OR* odds ratio, *CI* confidence interval, *CR* complete remission, *BMI* body mass index, *HOMA-IR* homeostatic model assessment of insulin resistance.

**Table 6:** Independent factors linked to complete remission of T2DM 2 years after LSG using logistic regression.

	B	*p* value	OR (95% CI)
Age ≤ 45 vs. > 45	1.929	0.001	6.9 (2.1–22.2)
Duration of DM ≤ 5 vs. > 5 years	2.808	< 0.001	16.6 (4.8–57.9)
Oral hypoglycemics Single vs. Multiple	2.079	0.006	8.0 (1.8–35.4)
C-peptide > 2.72 vs. ≤ 2.72 ng/mL	5.643	< 0.001	282.2 (23.6–3377.5)
HOMA-IR ≤ 4.6 vs. > 4.6	3.579	< 0.001	35.8 (9.6–134.0)

*B* regression coefficient, *OR* odds ratio, *CI* confidence interval, *HOMA-IR* homeostatic model assessment of insulin resistance.

## Discussion

Several reports have confirmed bariatric surgery’s effectiveness in resolving type-2 diabetes with a diverse remission rate and/or improvement rate. Many studies have proposed predictors of remission of diabetes after weight-loss operations^19–27^. Others introduced scores for such predictors^28–33^. The majority of these studies included different types of bariatric procedures, particularly RYGB. Previous studies investigating SG cases involved a small sample size^21,34,35^ or omitted many important factors like age, HbA1c, and C-peptide^22,24,36^. Almost all studies followed up their patients for 1 year. The current research probably presents the largest series of patients treated with LSG who were followed up for 2 years.

The study demonstrated that predictive factors for T2DM complete remission at 2 years were age ≤ 45 years, duration of diabetes ≤ 5 years, use of a single oral antidiabetic, HbA1c ≤ 6.5%, HOMA-IR ≤ 4.6, C-peptide > 2.72 ng/mL, and BMI ≤ 40 kg/m^2^. On multivariate analysis, independent predictors of complete remission were younger age, shorter duration, single oral antidiabetic, lower HOMA-IR, and higher C-peptide. A combined marker of young age, short duration of DM, and low HOMA-IR provide a very good prediction of complete remission with 93% sensitivity and 82% specificity.

In the current study, complete remission occurred in 38% of patients in addition to 10.6% partial remission. Metabolic failure is observed in only 6.2% of cases. A recent meta-analysis in the United Kingdom reported a rate of diabetes remissions of 94.5 per 1,000 person-years, an 18-fold increased chance for remission than matched controls^37^. The authors found a larger effect size in patients undergoing gastric bypass compared to SG. Malabsorptive procedures tend to have a better antidiabetic impact compared to restrictive procedures as SG^38^. It has been reported that RYGB results in 50–80% remission rate of T2DM^39–41^.

The explanation for resolution or improvement remains unclear, but decreased energy intake and weight loss probably significantly contribute to this process. However, early resolution of diabetes within 1 week of surgery indicates that weight loss per se cannot explain the entire mechanism^33,42^. The effect of restrictive procedures as SG may be attributed to the immediate and severe caloric restriction that forces the human body to use internal energy sources^43^. Thus, ectopic fat from the hepatic tissue and other stores are mobilized and utilized^44^. A reduction in liver fat content normalizes hepatic insulin sensitivity by improving fasting plasma glucose^43^. These findings are supported by observing liver volume and liver fat content reduction after a low-calorie diet before bariatric surgery^45–47^. Moreover, after 1 week of restricted energy intake, normalization of β-cell function has been demonstrated^48^. Therefore, food restriction can sensibly explain the rapid postoperative metabolic improvement.

However, SG can be viewed as more than a restrictive procedure. It has been followed by decreased ghrelin secretion and increased GLP-1 synthesis, changes similar to those reported after in RYGB^49^. It was suggested that the balance between ghrelin and GLP-1 might be the key to improved glucose homeostasis^50^. Numerous studies have verified increased levels of bile acids after SG that could lead to ameliorations in insulin sensitivity, incretin secretion, and postprandial glycemia^51,52^.

The predictive factors found in the current study go in accordance with the shared denominators of diabetes remission in previous studies. Many studies proposed younger age^19,25–27,53^, shorter duration of DM^20,23,26,27,54,55^, lower HbA1c^19,20,23,25,33^, no preoperative insulin use^26,55,56^. Like the present study, others found a higher rate of complete remission with lower insulin resistance expressed as lower levels of HOMA-IR^57,58^. In some studies, poor control of DM with lower preoperative C-peptide levels was linked to poor control of diabetes after bariatric surgery^25,59,60^.

In obesity-associated T2DM, decreased insulin sensitivity is the first lesion, ensued by elevated insulin levels. Hyperglycemia arises because of β-cell failure to synthesize sufficient insulin. T2DM develops when pancreatic β-cell dysfunction follows in the face of decreased insulin sensitivity^61^. Hepatic insulin resistance is mainly implicated in elevated blood glucose levels and overt diabetes^20^. Insulin resistance with caloric overload leads to hepatic steatosis causing hepatic insulin resistance. Consequently, insulin fails to suppress liver glucose production leading to hyperglycemia^43^, and the process is self-exacerbating. Higher HOMA-IR levels indicating greater insulin resistance can be markers of less chance of diabetic remission after bariatric surgery.

It sounds logical that less preoperative β-cell dysfunction is a determinant of diabetes remission following surgery. This is because the steady deterioration of β-cell function is an aspect of the natural history of diabetes^25^. The current study found that a C-peptide level < 2.7 ng/mL is an independent factor predicting complete remission of DM. C-peptide is considered a more accurate evaluation of pancreatic β-cell function than insulin. A large percentage of insulin synthesized by the pancreas is degraded during the first pass hepatic metabolism compared to a negligible C-peptide amount^62^. Older age and longer duration of diabetes are consequently associated with lower residual β cell mass^63,64^.

Prediction models of diabetes remission were previously suggested for more practical implementation in clinical practice. For example, the DiaRem score included four variables for accurate remission prediction, namely the use of insulin, age, HbA1c, and type of antidiabetic medication^65^. The need for insulin therapy was the strongest indicator in this scoring system. Later studies ensured external validation of this score in independent populations^66,67^. ABCD score determined four factors; age at surgery (A), baseline BMI (B), C-peptide level (C), and diabetes duration (D) to construct a 0 to 10 scoring system^31^.

Almost all studies and predicting scores share common factors that can be categorized into two groups, disease status and preoperative management characteristics. Collectively, a longer duration and more progressive disease requiring more aggressive treatment allocate the patient fewer odds of diabetic remission. Careful preoperative assessment of these factors may guide a better selection of bariatric surgery candidates who will benefit from resolving the serious comorbidity T2DM alongside weight reduction.

Although the literature reported much data regarding the improvement of T2DM after LSG, to our knowledge, only a few studies addressed the factors linked to complete and partial remission, improvement, or unchanged status of T2DM after LSG. Besides, the reasonable number of cases with an acceptable follow-up period is a strength point of this study. The limitations of our study are mainly its nature as a single-center study. This raises the possibility of selection bias and difficulty of generalization to the entire bariatric population.

It should be noted that patient who did respond to sleeve may proceed malabsorptive procedures such as single anastomosis gastric bypass or Roux en-Y gastric bypass. Duodenal switch as well as (Single anastomosis duodeno–ileal bypass) SADI are not commonly practiced in our center.

In conclusion, we found that independent predictors of complete remission of T2DM after LSG were younger age, shorter duration, single oral antidiabetic, lower HOMA-IR, and higher C-peptide. A combined marker of young age, short duration of DM, and low HOMA-IR provide a perfect prediction of complete remission with 93% sensitivity and 82% specificity. It is sensible to say that this work can help recognize cases that are most likely to avail from SG regarding the extent of T2DM remission.
